# Optimizing additive combinations to improve peanut vine silage fermentation quality and feed efficiency for sustainable livestock production

**DOI:** 10.5713/ab.250448

**Published:** 2025-11-10

**Authors:** Lijie Zhang, Wan Xiang, Yuliang Chen, Mengqi Tang, Wenjuan Li, Liyang Zhang, Tong Fu

**Affiliations:** 1 Henan International Joint Laboratory of Nutrition Regulation and Ecological Raising of Domestic Animal, College of Animal Science and Technology, Henan Agricultural University, Zhengzhou, China

**Keywords:** Feed Conversion Efficiency, Microbial and Enzymatic Additives, Nonconventional Forage Eesources, Ruminant Production Systems, Silage Fermentation Dynamics

## Abstract

**Objective:**

Peanut vine is a widely available agricultural by-product with high nutritional value, but its utilization is limited by poor ensiling characteristics. The purpose of this study was to improve the fermentation quality of peanut vine silage by using lactic acid bacteria (LAB), compound enzymes, and molasses both separately and in combination, and to evaluate their effects on growth performance and metabolic indicators in fattening *Hu* sheep.

**Methods:**

Peanut vine was treated with different levels of LAB, enzymes, and molasses to determine optimal dosages. The optimal combination of 2 g/t LAB, 200 mL/t enzyme preparation, and 10 kg/t molasses was identified based on fermentation characteristics. Treatments included a control (no additive), single additives, and the combined treatment. After 60 d of ensiling, silage pH, lactic acid, ammonia-N, fiber degradation, and bacterial community structure were analyzed. A 56 d feeding trial was subsequently conducted with 56 *Hu* sheep (28.4±1.3 kg), randomly assigned to two groups: peanut vine hay and peanut vine silage. Growth performance and serum biochemical parameters were assessed.

**Results:**

The optimal additive combination significantly improved fermentation by reducing pH (from 4.74 to 4.36), ammonia-N/total nitrogen (from 2.82% to 1.50% dry matter [DM]) and detergent fiber contents (neutral detergent fiber from 43.9% to 41.3% and acid detergent fiber from 34.6% to 32.2%), while increasing lactic acid concentration (from 3.55% to 5.00% DM). Microbial analysis revealed a higher relative abundance of *Lactobacillus plantarum* and increased microbial diversity. In the animal trial, no significant differences were found in average daily gain, DM intake, or feed conversion ratio between groups. However, sheep fed silage showed lower blood urea nitrogen and higher serum triglyceride concentrations, indicating improved nitrogen and lipid metabolism.

**Conclusion:**

Peanut vine silage treated with LAB, enzymes, and molasses improves fermentation quality and supports comparable growth performance to hay while improving nutrient metabolism in fattening *Hu* sheep. This approach provides a sustainable strategy for converting legume crop residues into valuable ruminant feed.

## INTRODUCTION

Mutton plays a crucial role in sustainable livestock production, and the growing global demand has prompted efforts to optimize feed resources and enhance the utilization of agricultural byproducts within circular farming systems. Global mutton production has steadily increased from 11.28 million tons in 2000 to 16.03 million tons in 2020, with China contributing approximately 30% of the total output. However, challenges such as the scarcity of roughage and the limited availability of high-quality forages highlight the need to explore alternative, locally available feed resources [1,2]. Peanut (*Arachis hypogaea* L.) is an important oil- and protein-producing crop cultivated worldwide [3]. During harvest, large quantities of peanut vines are produced, which, if not properly utilized, become potential agricultural waste. Peanut vine has a high nutritional value, characterized by its relatively high crude protein (CP) content (~12%), and higher digestibility of dry matter (DM), neutral detergent fiber (NDF), and acid detergent fiber (ADF) compared with alfalfa [4]. Although sometimes used as green fodder for ruminants, the high moisture content of fresh peanut vines leads to rapid spoilage, limiting their preservation and long-term utilization as forage [4]. Therefore, appropriate conservation methods are required to extend the shelf life and feeding value of peanut vines.

Ensiling is a widely adopted method for preserving forage crops through lactic acid (LA) fermentation under anaerobic conditions [5]. During this process, epiphytic or inoculated microorganisms convert water-soluble carbohydrates (WSC) into organic acids, thereby decreasing the pH, inhibiting spoilage organisms, and enhancing aerobic stability and overall silage quality [6]. However, peanut vines, being a leguminous resource with low WSC content, present challenges to efficient silage fermentation [7]. Furthermore, quality losses often occur during ensiling, which may reduce the feeding value of the final product [7]. To address these challenges, the use of silage additives such as lactic acid bacteria (LAB) inoculants (e.g., *Lactobacillus plantarum*, yeast, and *Bacillus* spp.), sugar sources (e.g., molasses), and fibrolytic enzymes (e.g., cellulase, hemicellulase, and pectinase) has become increasingly common for improving fermentation efficiency and nutrient preservation [8]. Previous studies have shown that LAB inoculation significantly enhances the quality of sugarcane silage [9], while molasses improves the fermentation quality of cassava leaf silage by modulating the microbial community and promoting fiber degradation [10]. Similarly, Chen et al [11] reported its potential to improve silage fermentation and fiber degradation. De Souza and Kawaguti [12] found that enzyme addition could break down plant cell walls and improve silage quality, and Kuo et al [13] demonstrated that hydrolyzing orange peel with cellulase and pectinase increased the content of fermentable sugars and improved fermentation performance.

In parallel, meat consumption in China has grown significantly, increasing from 18.2% in 2014 to 25.3% in 2022 [14]. Among various meat types, mutton has steadily gained popularity due to its low fat content and high nutritional value, leading to its expanding role in the traditional meat sector [15]. This growing demand underscores the importance of developing sustainable, cost-effective feed resources. Peanut vine, with its high nutritive value and abundance, shows great potential as a forage material in sheep production systems. However, few studies have systematically evaluated the effects of different types or combinations of additives on the fermentation quality of peanut vine silage. Therefore, in this study, we first applied different doses of LAB, complex enzyme preparations, and molasses individually to explore their optimal dosages for peanut vine ensiling. Furthermore, combinations of these additives were tested to investigate their synergistic effects on fermentation quality. Finally, a feeding trial with fattening *Hu* sheep was conducted using the treated silages to provide a scientific basis and practical recommendations for the effective utilization of peanut vine and silage fermentation additives in mutton production systems. We hypothesized that the combined application of LAB, enzyme preparations, and molasses would synergistically enhance peanut vine silage fermentation quality and promote better nutrient utilization and metabolic efficiency in *Hu* sheep.

## MATERIALS AND METHODS

### Ensilage preparation

Peanuts were cultivated at the experimental base of Henan Agricultural University, located in Zhengan town, Zhongmu County. Peanut vines were harvested at full maturity stage of peanuts, with a residual stubble height of 3–5 cm. After harvesting, the vines were cut into 3–5 cm sections using a hay cutter, and the chopped materials were placed on a clean plastic sheet as raw material for silage preparation.

A randomized block design was employed, consisting of four treatment groups (Table 1). (1) LAB group: *Lactobacillus plantarum* (≥5.0×10^10^ cfu/g) and *Micrococcus lactis* (≥1.5×10^10^ cfu/g) (Raman Animal Nutrition) were applied at 1 g/t (L1), 2 g/t (L2), 3 g/t (L3), and 4 g/t (L4). Based on the viable counts of the product, these inoculation levels were above the generally accepted minimum requirement for successful ensiling. The control (CK2) received an equal volume of distilled water. (2) Enzyme preparation group: a compound enzyme (Xia Sheng Industrial; cellulase: cellobiase: pectinase = 2:1:1, with respective activities of 2,500 U/mL, 1,000 U/mL, 15,000 U/mL) was applied at 50 mL/t (EP1), 100 mL/t (EP2), 150 mL/t (EP3), and 200 mL/t (EP4), with the control (CK1) receiving distilled water. (3) Molasses group: molasses (70% purity, 41% sugar content) was added at 5 kg/t (M1), 10 kg/t (M2), 15 kg/t (M3), and 20 kg/t (M4), with CK1 as the control. (4) Combined additive group: treatments included M-EP (10 kg/t molasses+200 mL/t enzyme), M-L (10 kg/t molasses+2 g/t LAB), EP-L (200 mL/t enzyme+2 g/t LAB), and M-EP-L (10 kg/t molasses+200 mL/t enzyme+2 g/t LAB), with CK3 as the control. All groups had 5 replicates, including 1 spare sample, except the combined additive group, which had 20 samples per replicate. Additives were dissolved in distilled water and sprayed evenly onto chopped peanut vines at 25 mL/kg (fresh matter); control group received water only. After thorough mixing, each replicate consisting of 1,000 g sealed in polyethylene fermentation bags, vacuum-sealed, and stored in a cool, dry place. Clean plastic sheets were used to prevent cross-contamination. Single-additive samples were analyzed after 60 d, while mixed-additive samples were tested at 3, 7, 14, 30, and 60 d.

The peanut vine silage used in the feeding experiment with *Hu* sheep was prepared from fresh peanut seedlings sourced from Shantang Town, Xunxian County, Henan Province. The whole plants were chopped into 3–5 cm sections and supplemented with 10 kg/t molasses, 200 mL/t enzyme preparation, and 2 g/t LAB. The mixture was packed into 600 kg round bales using a wrapping machine, fermented for 45 d, and then opened for feeding. Additionally, peanut vine hay purchased from Zhengyang County, Zhumadian City, Henan Province, was ground and de-dusted before use.

### Assessing silage for chemical composition and quality of fermentation

#### Assessing silage for sensory evaluation

Based on the color, odor and structural characteristics of peanut vine silage after fermentation, the German Agricultural Society scoring method was used to evaluate its quality. Silage was classified into four quality grades: poor (0–4 points), fair (5–9 points), good (10–15 points), and excellent (16–20 points) [16].

#### Assessing of silage fermentation quality

Fresh fermented peanut vine samples (20 g) were diluted with 180 mL of distilled water and stored at 4°C for 24h. The extracts were then filtered through sterilized double-layer gauze. The pH of the filtrate was measured using a PB-10 pH meter (Sartorius). The concentrations of LA, acetic acid (AA), propionic acid and butyric acid were determined using an ion chromatography system (ICS-3000; Dionex Corporation) equipped with an IonPac AS11-HC column (Φ4, L250 mm). The column temperature was 30°C, with a flow rate of 1.2 mL/min and an injection volume of 50 μL. NH_3_-N content was determined using the phenol-hypochlorite colorimetric method described [16].

#### Assessing of silage microbial population

Fresh silage samples (20 g) were diluted with 180 mL of sterile 0.9% saline solution and shaken for 30 min. Serial dilutions ranging from 10 to 10^6^ were prepared. LAB were cultured on de Man, Rogosa, and Sharpe (MRS) agar supplemented with 0.1 g/L cycloheximide and incubated at 37°C for 48 h. Microbial populations were quantified by plate counting, and results were expressed as colony-forming units (CFU) per gram of silage.

#### Assessing of silage chemical composition

The DM content of silages before and after ensiling was determined by drying at 65°C to a constant weight in a fan-assisted oven. Sample was ground by passing it through at 1-mm mesh sieve in a knife mill (YB700; Yunbang Instrument) and stored for subsequent analyses. The CP content was measured using an automatic distillation and titration system (K9860; Haineng Instrument) following the procedure by Krishnamoorthy et al [17]. The NDF and ADF contents were analyzed using an ANKOM 220 Fiber Analyzer (ANKOM Technology) with sodium sulfide and α-amylase, according to the official AOAC method [18]. The WSC content was determined by the phenol-sulfuric acid method [19].

### Bacterial community analysis

#### DNA extraction and polymerase chain reaction amplification

Total microbial genomic DNA was extracted from 32 silage samples using the E.Z.N.A. soil DNA Kit (Omega Bio-tek) following the manufacturer’s instructions. DNA concentration and purity were assessed using 1.0% agarose gel electrophoresis and a NanoDrop2000 spectrophotometer (Thermo Fisher Scientific). Extracted DNA was stored at −80°C until further analysis. The hypervariable region V3-V4 of the bacterial 16S rRNA gene were amplified using primer pairs 338F (5’-ACTCCTACGGGAGGCAGCAG-3’) and 806R(5’-GGACTACHVGGGTWTCTAAT-3’) by T100 Thermal Cycler polymerase chain reaction (PCR) thermocycler (Bio-Rad Laboratories). The PCR reaction mixture including 4 μL 5×Fast Pfu buffer, 2 μL 2.5 mM dNTPs, 0.8 μL each primer (5 μM), 0.4 μL Fast Pfu polymerase, 10 ng of template DNA, and ddH_2_O to a final volume of 20 μL. PCR amplification cycling conditions were as follows: initial denaturation at 95°C for 3 min, followed by 27 cycles of denaturing at 95°C for 30 s, annealing at 55°C for 30 s and extension at 72°C for 45 s, and single extension at 72°C for 10 min, and end at 4°C. The PCR product was extracted from 2% agarose gel and purified using the PCR Clean-Up Kit (YuHua) according to manufacturer’s instructions and quantified using Qubit 4.0 (Thermo Fisher Scientific).

#### Sequencing and data analysis

An Illumina MiSeq PE300 platform (Illumina) was used to sequence the PCR product. After purification and quantification, FLASH (ver. 1.2.11) was used to assemble raw reads, with the QIIME quality control process (ver. 1.7.0) used to exclude low quality reads and UCHIME algorithm used to remove chimeric sequences and obtain final effective tags. Operational taxonomic units (OTUs) with an overall similarity of 97% were collected with a UPARSE pipeline. QIIME software (ver. 1.7.0) was used to determine the Shannon index and Chao richness estimator, as well as Good’s coverage and other alpha diversity indexes. Taxonomy classification was conducted using the Ribosomal Database Project (RDP) and SILVA database (70% of confidence threshold) to determine community structure (phylum and genus). Principal component analysis (PCA) based on weighted UniFrac distances was performed and visualized using R software. Heatmaps showing Spearman correlation coefficients between the bacterial community and fermentation parameters were generated with the Vegan package in R.

### Experiment of fattening *Hu* sheep with wrapped peanut vine silage

#### Animals, experimental design, and dietary treatments

The feeding trial was conducted at the Yuemeihe Agriculture and Animal Husbandry Development.

Fifty-six healthy weaned *Hu* sheep (120 days old; initial body weight (BW) = 28.4±1.3 kg) were blocked into two groups (28 animals each, with equal numbers of males and females) based on BW, and randomly assigned to eight identical pens (four pens per block; seven sheep per pen). Each of the two experimental diets was allocated to one pen per block. The total mixed rations (TMR) were formulated with different forage sources (Table 2): (1) a dry peanut vine-based diet, and (2) a peanut vine silage-based diet. A14-day adaptation period was provided during which all sheep were fed the dry peanut vine diet. From d 10 of adaptation, a gradual dietary transition was implemented by replacing 20% of the dry peanut vine with silage every 2 d. The transition was completed by d 14, and the feeding trial commenced immediately thereafter, lasting for 56 d. All animals had *ad libitum* access to TMR and water, and were fed twice daily at 8:00 and 17:00.

#### Sample collection and measurements

The TMR and refusals were weighed and sampled daily. BW was recorded before morning feeding for three consecutive days at the start of the trial and again every 28 d. Dry matter intake (DMI), average daily gain (ADG), and ratio of feed to gain (F/G) were calculated accordingly. Blood samples were collected from the jugular vein using 10 mL vacuum tubes (without anticoagulants) in the early morning before feeding on d 0, 28 and 56. Samples were centrifuged at 1,500×g for 15 min, and the supernatant serum was collected using disposable straws and stored in Eppendorf tubes at −20°C for further analysis. Serum biochemical indices were determined using an automated biochemical analyzer (Beckman Coulter Au5800).

### Statistical analyses

All statistical analyses were performed using SPSS 22.0 software (IBM). Data were first tested for normality using the Shapiro–Wilk test and for homogeneity of variance using Levene’s test. A one-way analysis of variance (ANOVA) was then conducted, with treatment or diet as the fixed effect, according to the following model:


(1) $$Y_{ij}=\mu +T_{i}+{ɛ}_{ij},$$

where *Y_ij_* is the observed value, *μ* is the overall mean, *T_i_* represents the effect of treatment (for silage fermentation parameters) or diet (for the animal trial), and *ε_ij_* is the residual error. Each silo was considered the experimental unit for silage parameters, and each pen of seven sheep was considered the experimental unit for the animal trial. When a significant main effect was detected, mean comparisons were performed using Duncan’s multiple range test. Results are expressed as means± standard deviation (SD) and statistical significance was declared at p<0.05.

## RESULTS

### Performance

#### Effects of different additives and levels on the sensory evaluation of peanut vine silage

As shown in Table 3, after 60 d of ensiling, the CK received a sensory evaluation score corresponding to grade 2. The addition of LAB, complex enzyme preparations, and molasses improved the visual, olfactory, and structural characteristics of the silage. Notably, molasses enhanced the acidic aroma of the silage.

#### Effects of different additives and levels on the fermentation quality of peanut vine silage

Table 4 presents the fermentation characteristics of silage treated with different additives and concentrations. After 60 d, silage treated with high concentrations of compound enzyme preparations (EP3, EP4) exhibited lower pH compared to those treated with low concentrations (p<0.05). Similarly, pH was reduced in molasses-treated groups relative to the control (p<0.05).

The NH_3_-N/TN content decreased in silage treated with LAB compared to the control group (p<0.05). High levels of enzyme preparation (EP3, EP4) also lowered NH_3_-N/TN content (p<0.05). Furthermore, increasing molasses concentrations gradually reduced NH_3_N/TN, with M2, M3, and M4 treatments showing decreases (p<0.05).

LA content increased in the L3 and L4 groups compared to CK2 (p<0.05). A similar increase was observed in the EP3 and EP4 groups, which higher LA levels than CK1 and EP1 (p<0.05). Molasses addition also enhanced LA production (p<0.05).

AA content was lower in the L2 and L3 groups compared to CK1 (p<0.05). Increasing molasses concentration led to a reduction in AA content, with M2, M3 and M4 treatments showing lower levels than CK1 (p<0.05).

The number of LAB were higher in silage treated with low concentrations of LAB (L1, L2, L3) than in CK2 (p<0.05). However, high concentrations of compound enzyme preparations (EP2, EP3, EP4) reduced LAB counts compared to CK1 (p<0.05). Similarly, LAB counts in molasses-treated groups were lower than in CK1 (p<0.05).

#### Effects of different additives and levels on the chemical composition of peanut vine silage

As shown in Table 5, after 60 d of fermentation, the DM content of silage in the L3 group (3 g/t LAB) was higher than in CK2 (p<0.05). Silage treated with compound enzyme preparations also showed higher DM content than CK1 (p<0.05), while no difference was observed between molasses-treated groups and CK1.

The CP content was increased in silage treated with high levels of LAB (L2, L3, L4) compared to CK2 and L1 (p<0.05). Likewise, high concentrations of enzyme preparations (EP3, EP4) enhanced CP content compared to CK1 and lower concentration groups (EP1, EP2) (p<0.05). Silage treated with 5 kg/t (M1), 10 kg/t (M2) and 20 kg/t (M4) of molasses also showed higher CP content than CK1 (p<0.05).

LAB addition decreased NDF content compared to CK2 (p<0.05). While ADF was lower in the L1, L2 and L4 groups than in CK2 (p<0.05). Similarly, enzyme preparation treatments reduced both NDF and ADF contents compared to CK1 (p<0.05). The addition of molasses also decreased both NDF and ADF levels in all treatment groups (p<0.05).

No change in soluble sugar content was observed with the addition of LAB. However, the EP4 group (high enzyme concentration) and all molasses-treated groups exhibited higher soluble sugar levels compared to CK (p<0.05).

### Effects of mixed additives on peanut vine silage at different fermentation stages

#### Sensory evaluation

As shown in Table 3. After 60 d of fermentation, silage was unsealed for sensory evaluation. The control group received a Level 2 rating, while all mixed additive treatments achieved Level 1. Among them, the M-EP-L group attained the highest score, indicating superior sensory characteristics and appearance of silage compared to other treatments.

#### Fermentation quality

**Figure 1:**
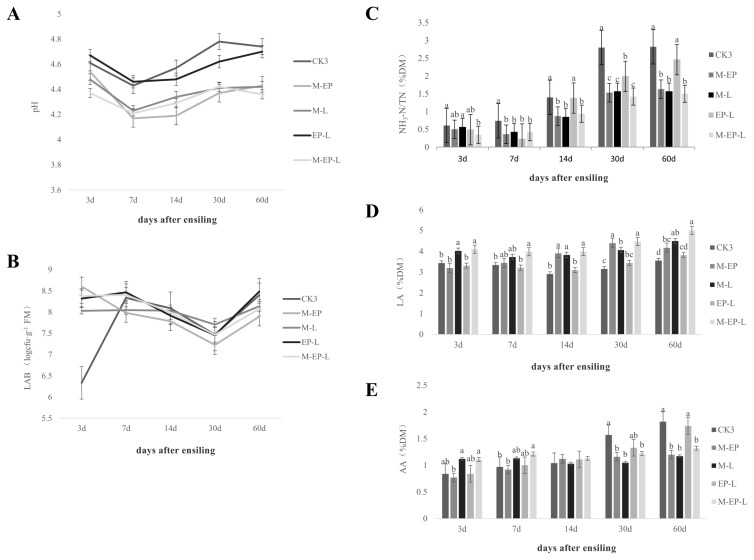
Fermentation characteristics of peanut vine silage treated with different additive combinations after 60 d of ensiling. (A) pH value; (B) *Lactobacillus* count; (C) NH_3_-N/TN ratio; (D) lactic acid content; (E) acetic acid content. Different lowercase letters (^a–d^) above the bars indicate significant differences among treatments at the same time point (p<0.05).

Fermentation dynamics across treatments are shown in Figure 1. On d 3, the M-EP-L group had the lowest pH (p<0.05). The CK3 group showed the highest pH, which was not different from the EP-L group, but was higher than the M-EP and M-L groups (p<0.05). Across all time points, pH in CK3 and EP-L groups remained higher than in the M-EP-L group (p<0.05).

On d 3, LAB counts in the M-EP group were higher than in CK3 (p<0.05), but from d 7 onward, LAB counts in the M-EP group were lower than those in the M-L, M-EP-L, and CK3 groups (p<0.05).

The NH_3_-N/TN ratio in the CK3 and M-L groups was higher than in the M-EP-L group on d 3 (p<0.05). By d 7, the CK3 group had the highest ratio, and at d 14, 30, and 60, NH_3_-N/TN ratio in CK3 and EP-L remained higher than the other groups (p<0.05).

LA content in the M-EP-L group was higher than in the M-EP group on d 3 (p<0.05). On d 7, M-L was higher LA content than EP-L (p<0.05), while M-EP-L had the highest content on d 60 (p<0.05). For AA, no differences appeared on d 3 and 14. On d 7, M-L was higher than the M-EP and EP-L (p<0.05). While on d 30, CK3 was higher AA than the M-EP, M-L, and M-EP-L groups, and by d 60, CK3 and EP-L still exhibited the highest AA content among all groups (p<0.05).

#### Chemical composition

As shown in Table 6, the DM content peaked on d 3. On d 3, 7, and 30, CK3 and EP-L had lower DM than the other treatments (p<0.05) while no differences were observed at d 14 and 60. On d 3, the CP content in M-EP was higher than in CK3 (p<0.05). On d 7 and 14, the CP content in CK3 and M-L was lower than the other groups (p<0.05). On d 30, the CP content in EP-L was lower than that in CK3 and M-EP-L (p<0.05). By d 60, the CP content in M-EP was lower than in CK3 and M-EP-L, while EP-L was higher CP content than M-L and M-EP-L (p<0.05).

The NDF and ADF contents varied across groups and time points. On d 3, CK3 had the lowest values, lower than those in M-L and EP-L (p<0.05), and EP-L had lower ADF than M-L (p<0.05). On d 7, EP-L had higher NDF than M-EP and CK3 (p<0.05), and M-EP had higher ADF (p<0.05). On d 14, M-EP-L had the lowest NDF, while CK3, M-L, and EP-L had the lowest ADF (p<0.05). On d 30, there were no differences. By d 60, CK3 and EP-L had higher NDF and ADF contents than M-EP-L (p<0.05).

Soluble sugar content also varied. On d 3, M-EP and M-EP-L had higher levels than CK3 and EP-L (p<0.05), with EP-L showing the lowest values at most time points. On d 7, M-L and M-EP-L were higher than CK3 (p<0.05). By d 14, M-EP-L remained the highest, followed by M-L, which was higher than EP-L (p<0.05). On d 30, M-EP-L was higher than CK3, M-EP, and EP-L, while EP-L was lower than M-L and M-EP-L (p<0.05). On d 60, M-EP-L showed the highest soluble sugar content, while EP-L had the lowest (p<0.05).

#### Microbial community analysis

**Figure 2:**
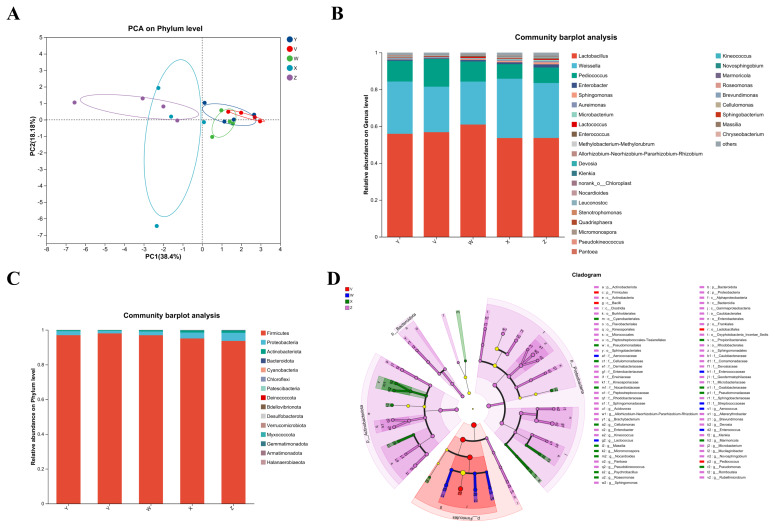
Bacterial community composition of peanut vine silage supplemented with different additive combinations after 60 d of ensiling. (A) Principal coordinate analysis (PCA) of bacterial communities; (B) Composition at the phylum level; (C) Composition at the genus level; (D) LEfSe analysis (LDA score>2) showing significantly different taxa among treatments. V = CK3 (control); W = M-EP; X = M-L; Y = EP-L; Z = M-EP-L. LEfSe, linear discriminant analysis effect size; LDA, linear discriminant analysis.

The number of high-quality sequences per sample ranged from 46,237 to 67,624, with an average of 57,736 sequences per sample. Sequencing depth was sufficient, as all samples showed coverage values above 99%. Among the treatments, the M-EP-L group exhibited the highest number of OTUs (approximately 207), accompanied by the greatest ACE and Chao1 estimates. In general, silages treated with combined additives (M-EP, M-L, EP-L, and M-EP-L) showed higher OTUs, ACE, Chao1, and Sobs indices than the control (CK3), indicating enhanced bacterial richness and diversity. Principal coordinates analysis (PCoA) based on weighted UniFrac distances revealed clear clustering of bacterial communities among treatments (Figure 2A). At the phylum level, Firmicutes and Proteobacteria were dominant across all groups, while the relative abundance of Bacteroidota varied slightly among treatments (Figure 2B). At the genus level, *Lactobacillus*, *Pediococcus*, and *Enterococcus* were enriched in silages supplemented with additives, whereas *Pantoea* predominated in the control group (Figure 2C). Furthermore, LEfSe analysis identified specific bacterial taxa that significantly discriminated between treatments, including enrichment of *Lactococcus* and *Pediococcus* in the LAB-treated silage, *Devosia* in the enzyme-treated group, and *Sphingomonas* in the molasses-enzyme combination (Figure 2D). These findings suggest that additive supplementation reshaped the bacterial community by selectively promoting beneficial LAB while suppressing undesirable taxa.

### Effects of feeding peanut vine silage on *Hu* sheep

#### Growth performance

As shown in Table 7, no differences were observed in ADG or DMI between *Hu* sheep fed peanut vine hay and those fed peanut vine silage throughout the experimental period.

#### Blood biochemical parameters

As shown in Table 8, the BUN levels on d 56 were higher in the control group than in the test group (p<0.05). In contrast, triglyceride (TG) levels on d 28 and 56 were lower in the control group compared with the test group (p<0.05).

## DISCUSSION

The results of the sensory evaluation and quality grading indicated that the sensory properties and quality class of peanut vine silage with various additives were superior to those of the CK group. This improvement can be attributed to the additives, which reduce the acidity of the silage, inhibit the growth of harmful bacteria, and minimize nutrient loss [20]. During fermentation, volatile organic compounds (VOCs) are produced, contributing to acidity and aroma that enhance the odor of the silage. Liu et al [21] found that adding LAB positively influenced the sensory quality by producing flavor compounds in the pickle samples. Similarly, Zhang et al [22] reported that lactobacillus additives altered the odor of silage by modifying the microbial community. Ebrahimi et al [23] demonstrated that combining LAB with cellulase significantly improved the quality of the oil palm frond silage. Xing et al [24] found that enzyme additives increased the LA content and enhanced the fermentation quality of sorghum straw silage to varying degrees. The combined addition of LA, enzyme preparations, and molasses increased the LA content, decreased the pH value, and facilitated the smooth progression of peanut vine silage fermentation, thereby protected proteins from degradation and reduced the production of undesirable odors. There was a clear negative correlation between pH and LA content, as lower pH corresponded to higher LA levels. The inoculation levels applied in this study were sufficient to ensure effective fermentation, as they provided viable counts well above the threshold generally required for silage fermentation. In contrast, undesirable microorganisms during the ensiling process can produce off-odors and harmful substances, leading to deterioration in color and reducing animals’ willingness to consume silage. Compound additives were particularly effective in rapidly lowering pH, inhibiting undesirable microbial growth, and creating an oxygen-free environment, which slowed oxidative reactions and preserved the structure and color of the feed.

The addition of different combinations of additives to peanut vine silage improves the apparent characteristics of the silage. During the silage process, the types and quantities of dominant bacteria, such as LAB, directly influence the fermentation process and determine whether LAB should be added to the silage [25]. The conversion efficiency of heterofermentative LAB in producing LA is only 17%–50% of that of homofermentative strains, resulting in nutrient loss in the feed and the conversion of LA into AA [26]. The addition of molasses to silage effectively promotes LA fermentation, reduces the pH of silage, inhibits harmful bacteria activity, minimizes protein degradation, improves fermentation quality, and reduces nutrient losses during fermentation [11]. The rate of pH decline is an important indicator of microbial activity and silage fermentation. Throughout the silage process, the pH values of the CK3and EP-L groups were higher than those of other treatment groups. The combination of molasses, LAB, and enzyme preparations resulted in the most significant pH reduction across treatments. On d 7 of the experiment, the pH of all groups showed a decreasing trend, with the M-Land M-EP-L groups exhibiting the largest decreases. From d 14 to 60, the pH value gradually increased. During the silage process for peanut seedlings, the pH values of each group varied at the same time points. Desta et al [27] similarly observed that the main pH reduction occurred during the first 7 to 14 d of silage, with no significant further decrease as silage time extended. In this study, the pH changes among different combinations of additives followed the same trend: a decrease in the early stages, followed by a gradual increase, and then a slight reduction. The production of LA lowers the silage’s pH, making it acidic, which inhibits the growth of harmful microorganisms and preserves the silage’s quality and nutritional value [28]. Consistent with this study, Guo et al [29] found that the addition of LAB increased AA content in plateau silage maize. LAB fermentation also inhibits the heterogeneous decomposition of proteins, reducing the production of ammoniacal nitrogen [30]. The fermentation process of peanut vine silage was enhanced, partially degrading the fiber in the feed and generating soluble sugars as substrates, further improving silage fermentation quality [31].

The combination of LAB and enzymes synergistically enhanced fermentation by promoting LA production and fiber degradation, thereby improving the nutritional quality of the silage. In this experiment, after 60 d of silage fermentation, the DM content of peanut vine silage with added complex enzyme preparations was significantly higher than that of the control group. Since LAB cannot produce cellulolytic enzymes during silage fermentation, their addition alone does not reduce fiber content [32]. Xing et al [24] found that adding cellulase and LAB to sorghum and oil palm leaves reduced pH, increased LA content and *in vitro* DM digestibility, and decreased the contents of NDF and ADF, thereby improving silage fermentation quality. In this study, the addition of different levels of enzyme preparations significantly reduced the silage’s pH value. With increasing enzyme preparation levels, the pH value decreased progressively, with the EP3 and EP4 groups showing the most pronounced effects. The content of soluble carbohydrate decreased as silage time progressed; however, there was no significant difference in soluble sugar content between 30 and 60 d of silage. The addition of complex enzyme preparations promoted fiber degradation and generated WSC, which were utilized by LAB to produce LA [33]. The addition of molasses allowed silage to reach a stable state more quickly, inhibited nutrient degradation by harmful microorganisms, and reduced organic matter and nutrient losses. Besides molasses, the content of NDF and ADF decreased significantly, likely due to additives improving fermentation quality, producing more organic acids, and promoting the acidolysis of structural carbohydrates. Arbabi and Ghoorchi [34] reported that molasses significantly reduced NDF and ADF contents, indicating enhanced degradation of structural carbohydrates [34]. Consequently, the digestibility and nutrient absorption of silage by animals were improved.

Differences in the microbial community among raw materials can result from various factors, including climate, the chemical composition of materials, and regional factors [35]. The results of the bacterial community alpha diversity analysis indicated that the composite additive groups exhibited higher diversity. This could be attributed to their superior fermentation quality, which altered the microbial composition in peanut vine silage and increased its microbial richness. After 60 d of ensiling, Firmicutes and Proteobacteria were identified as the dominant phyla in peanut vine silage. Firmicutes are acid-hydrolytic microorganisms, while Proteobacteria contribute to the digestion of organic matter; both play critical roles in anaerobic environments [36]. It is well established that *Lactobacillus* plays a key role in enhancing LA production and reducing pH, becoming the predominant genus in high-quality silages. Similarly, when mulberry leaves were ensiled with *Lactobacillus casei* and sucrose, *Lactobacillus* emerged as the dominant genus across all treatments [37]. Moreover, combining LAB with 2% molasses significantly increased the relative abundance of desirable *Lactobacillus* while inhibiting the reproduction of undesirable microorganisms [38]. In this study, silages treated with additives exhibited improved fermentation quality compared to the CK silages.

Ensiling is an effective method for preserving fresh forages and plays a crucial role in enhancing the palatability and digestibility of agricultural products [39]. The silage is a feasible method for preserving peanut vine, providing high-quality feed for young Holstein bulls. In this study, peanut vine silage was used as a dietary component to feed fattening *Hu* sheep. Results showed that peanut vine hay and peanut vine silage had no significant effect on the feed intake of *Hu* sheep, nor did they lead to differences in growth performance. Blood biochemical indexes are valuable indicators of livestock production and nutritional status [40]. Serum blood urea nitrogen (BUN) concentration reflects protein and amino acid metabolism. Studies have shown that increasing dietary energy and nitrogen levels can enhance nitrogen utilization efficiency [41]. In this study, the CP content in the control group diet was higher than that in the experimental group, resulting in higher serum BUN concentrations in the control group. Serum TG, a fat metabolite, is decomposed by various tissues, and its concentration is closely linked to fat metabolism [42]. The experimental group had lower TG levels compared to the control group, suggesting that silage peanut vine may promote better dietary fat utilization. Other measured indexes remained within the normal range with no significant differences between the two groups. This indicates that neither peanut vine hay nor peanut vine silage had adverse effects on serum protein, blood glucose, or the immune system. In conclusion, peanut vine silage is a feasible and effective method for feed preservation, offering a practical alternative for maintaining the nutritional quality of roughage.

## CONCLUSION

This study demonstrated that the combined application of LAB, compound enzymes, and molasses exerted a synergistic effect that optimized peanut vine silage fermentation. The improvement was associated with enhanced microbial community structure, increased LA accumulation, and reduced proteolysis, suggesting that additive interactions play a pivotal role in regulating silage microbiology and chemistry. Importantly, the animal feeding trial confirmed that such silage can sustain growth performance while improving nitrogen and lipid metabolism in *Hu* sheep. Overall, these finding support the practical utilization of peanut vine as a valuable forage resource and provide new insights into host, microbe, and feed interactions in ruminant nutrition, offering a foundation for future mechanistic and applied studies in sustainable livestock production systems.

## Figures and Tables

**Table 1:** Experimental design of peanut vine silage under different additive treatments (*Lactobacillus*, enzyme preparation, and molasses, alone or in combination)

Items	*Lactobacillus* (g/t)	Enzyme (mL/t)	Molasse (kg/t)
CK2	0	0	0
L1	1	0	0
L2	2	0	0
L3	3	0	0
L4	4	0	0
CK1	0	0	0
EP1	0	50	0
EP2	0	100	0
EP3	0	150	0
EP4	0	200	0
CK1	0	0	0
M1	0	0	5
M2	0	0	10
M3	0	0	15
M4	0	0	20
CK3	0	0	0
M-EP	0	200	10
M-L	2	0	10
EP-L	2	200	0
M-EP-L	2	200	10

CK = control group without additives; L = lactic acid bacteria addition at different levels (1–4 g/t); EP = enzyme preparation addition at different levels (50–200 mL/t); M = molasses addition at different levels (5–20 kg/t). Combined treatments: M-EP = molasses+enzyme preparation; M-L = molasses+lactic acid bacteria; EP-L = enzyme preparation+lactic acid bacteria; M-EP-L = molasses+enzyme preparation+lactic acid bacteria.

**Table 2:** Composition and nutrient levels (dry matter [DM]%) of the experimental diets for *Hu* sheep fed peanut vine hay or peanut vine silage, and of the raw peanut vine material

Diet composition	Control group	Test group	Peanut vine (raw material)
Corn	25.0	25.0	
Wheat bran	6.5	6.5	
Soybean meal	15.5	15.5	
Premix	2.5	2.5	
Sodium bicarbonate	0.5	0.5	
Peanut vine hay	50.0	0.0	
Peanut vine silage	0.0	50.0	
Total	100	100	
Nutrient levels			
CP	14.62	13.62	10.50
NDF	32.99	32.76	35.80
ADF	20.96	20.63	26.95
EE	2.08	2.43	2.65
Ca	1.18	1.74	1.71
P	0.19	0.16	0.07

Control group = basal diet containing peanut vine hay; Test group = basal diet in which peanut vine silage replaced peanut vine hay.

Nutrient levels are expressed on a DM basis. Percentages of diet composition were rounded to one decimal place, ensuring a total of 100%.

Premix provided the following per kg of diet: VA, 10,000 IU; VD, 1,500 IU; VE, 125 IU; Fe, 1,600 mg; Cu, 200 mg; Mn, 1,300 mg; Zn, 1,300 mg; I, 8.75 mg; Se, 3.75 mg; Co, 3.75 mg; Ca, 14.5 g; P, 2 g; NaCl, 6.4 g.

CP, crude protein; NDF, neutral detergent fiber; ADF, acid detergent fiber; EE, ether extract; Ca, calcium; P, phosphorus.a

**Table 3:** Sensory evaluation of peanut vine silage treated with different additives and combinations after 60 d of ensiling

Items	Odor	Texture	Color	Total score	Grade
CK2	10	3	1	14	Level 2
L1	10	3	1	14	Level 2
L2	11	3	1	15	Level 1
L3	11	3	1	15	Level 1
L4	11	3	1	15	Level 1
CK1	10	3	1	14	Level 2
EP1	11	3	1	15	Level 1
EP2	11	3	2	16	Level 1
EP3	11	4	2	17	Level 1
EP4	11	4	2	17	Level 1
CK1	10	3	1	14	Level 2
M1	11	4	1	16	Level 1
M2	12	4	1	17	Level 1
M3	13	4	1	18	Level 1
M4	13	4	1	18	Level 1
CK3	10	3	1	14	Level 2
M-EP	11	4	2	17	Level 1
M-L	13	4	2	18	Level 1
EP-L	11	3	1	15	Level 1
M-EP-L	13	4	2	19	Level 1

Sensory evaluation was based on odor, texture, and color, with higher scores indicating better quality.

Total score was the sum of individual trait scores, and the final grade was assigned according to evaluation standards: Level 1 = high-quality silage; Level 2 = acceptable silage.

CK = control group without additives; L = lactic acid bacteria addition at different levels (1–4 g/t); EP = enzyme preparation addition at different levels (50–200 mL/t); M = molasses addition at different levels (5–20 kg/t); M-EP = molasses+enzyme preparation; M-L = molasses+lactic acid bacteria; EP-L = enzyme preparation+lactic acid bacteria; M-EP-L = molasses+enzyme preparation+lactic acid bacteria.

**Table 4:** Fermentation quality of peanut vine silage with different additive levels after 60 d of ensiling

Items	LAB group						Enzyme preparation group						Molasses group					
		
	CK2	L1	L2	L3	L4	p-value	CK1	EP1	EP2	EP3	EP4	p-value	CK1	M1	M2	M3	M4	p-value
pH	4.74± 0.03^a^	4.70± 0.04^a^	4.70± 0.02^a^	4.72± 0.02^a^	4.73± 0.03^a^	0.38	4.65± 0.05^a^	4.61± 0.12^a^	4.53± 0.07^a^	4.39± 0.09^b^	4.35± 0.07^b^	<0.001	4.65± 0.05^a^	4.47± 0.07^b^	4.31± 0.10^c^	4.29± 0.12^c^	4.23± 0.04^c^	<0.001
NH_3_-N/TN (%DM)	2.9± 0.2^a^	2.9± 0.1^a^	2.8± 0.1^ab^	2.8± 0.2^ab^	2.6± 0.0^b^	0.10	2.3± 0.3^a^	2.2± 0.3^a^	2.2± 0.1^a^	1.7± 0.3^b^	1.7± 0.2^b^	0.004	2.3± 0.3^a^	2.0± 0.6^ab^	1.6± 0.6^b^	1.5± 0.1^b^	1.5± 0.3^b^	0.05
Lactic acid (%DM)	2.8± 0.2^b^	3.2± 0.4^ab^	3.3± 0.5^ab^	3.5± 0.3^a^	3.7± 0.1^a^	0.02	3.1± 0.2^c^	3.0± 0.2^c^	3.4± 0.5^bc^	4.2± 0.5^a^	4.0± 0.7^ab^	0.01	3.1± 0.2^c^	3.8± 0.4^b^	3.4± 0.2^bc^	4.0± 0.2^b^	4.8± 0.8^a^	0.001
Acetic acid (%DM)	1.8± 0.1^a^	1.6± 0.1^ab^	1.5± 0.2^b^	1.5± 0.1^b^	1.6± 0.0^ab^	0.05	1.7± 0.0^a^	1.7± 0.1^a^	1.6± 0.3^a^	1.5± 0.1^a^	1.4± 0.2^a^	0.22	1.7± 0.0^a^	1.4± 0.4^ab^	1.0± 0.1^c^	1.2± 0.2^bc^	1.1± 0.1^bc^	0.002
Lactic acid bacteria (log cfu·g^−1^ FM)	7.2± 0.0^c^	7.3± 0.0^b^	7.3± 0.0^b^	7.4± 0.1^a^	7.2± 0.1^bc^	<0.001	7.2± 0.0^a^	7.2± 0.1^ab^	7.1± 0.2^b^	7.1± 0.1^b^	6.7± 0.0^c^	<0.001	7.2± 0.0^a^	6.8± 0.1^b^	6.7± 0.2^b^	6.8± 0.1^b^	6.7± 0.2^b^	<0.001

LAB group = silage treated with different levels of lactic acid bacteria (L1–L4 = 1–4 g/t); Enzyme preparation group = silage treated with different levels of enzyme preparation (EP1–EP4 = 50–200 mL/t); Molasses group = silage treated with different levels of molasses (M1–M4 = 5–20 kg/t).

CK = control group without additives.

Items measured included pH, ammonia nitrogen/total nitrogen (NH_3_-N/TN, % DM), lactic acid (% DM), acetic acid (% DM), and lactic acid bacteria counts (log cfu·g^−1^ FM).

a–c Within the same row, values followed by different lowercase letters differ significantly (p<0.05), while those with the same letter are not significantly different (p>0.05).

DM, dry matter.

**Table 5:** Effects of different additives and their levels on the nutrient composition (dry matter [DM]%) of peanut vine silage after 60 d of ensiling

Items	LAB group						Enzyme preparation group						Molasses group					
		
	CK2	L1	L2	L3	L4	p-value	CK1	EP1	EP2	EP3	EP4	p-value	CK1	M1	M2	M3	M4	p-value
DM	26.7± 0.3^b^	27.2± 0.4^ab^	27.3± 0.2^ab^	27.6± 0.8^a^	27.3± 0.6^ab^	0.17	25.3± 0.2^b^	25.7± 0.4^a^	25.8± 0.3^a^	26.1± 0.2^a^	26.0± 0.2^a^	0.01	25.3± 0.2^a^	25.2± 0.3^a^	25.3± 0.1^a^	26.3± 0.7^a^	25.8± 0.3^a^	0.01
CP	13.9± 0.2^c^	13.9± 0.2^c^	14.5± 0.1^ab^	14.7± 0.2^a^	14.3± 0.2^b^	0.003	14.6± 0.1^c^	15.1± 0.2^c^	15.2± 0.1^bc^	15.3± 0.1^ab^	15.4± 0.1^a^	<0.001	14.6± 0.1^c^	15.2± 0.1^a^	15.0± 0.5^ab^	14.7± 0.1^bc^	15.2± 0.2^a^	0.004
NDF	46.7± 0.6^a^	42.8± 0.8^b^	43.5± 0.8^b^	43.3± 1.1^b^	43.3± 1.2^b^	<0.001	44.3± 1.6^a^	42.6± 0.7^b^	41.8± 1.1^b^	41.5± 1.5^b^	42.5± 0.6^b^	0.03	44.3± 1.6^a^	39.9± 1.4^b^	40.1± 1.1^b^	39.6± 0.4^b^	39.6± 0.7^b^	<0.001
ADF	36.7± 0.5^a^	34.9± 0.9^bc^	33.9± 0.4^c^	35.8± 1.2^ab^	35.2± 1.3^bc^	0.01	35.5± 0.9^a^	33.7± 0.7^b^	33.9± 1.0^b^	33.3± 1.4^b^	34.6± 0.6^ab^	0.04	35.5± 0.9^a^	32.3± 1.1^b^	31.8± 0.5^b^	31.9± 0.5^b^	31.6± 0.5^b^	<0.001
WSC	0.77± 0.15	0.61± 0.19^a^	0.78± 0.02^a^	0.63± 0.03^a^	0.66± 0.10^a^	0.18	0.68± 0.05^b^	0.74± 0.06^b^	0.67± 0.15^b^	0.77± 0.11^b^	0.99± 0.09^a^	0.002	0.68± 0.05^d^	0.90± 0.06^b^	0.82± 0.10^bc^	0.76± 0.06^c^	1.04± 0.10^a^	<0.001

LAB group = silage treated with different levels of lactic acid bacteria (L1–L4 = 1–4 g/t); Enzyme preparation group = silage treated with different levels of enzyme preparation (EP1–EP4 = 50–200 mL/t); Molasses group = silage treated with different levels of molasses (M1–M4 = 5–20 kg/t).

CK = control without additives.

Measured items are expressed on a DM basis.

a–d Within the same row, values with different lowercase letters indicate significant differences (p<0.05), while those with the same letter are not significantly different (p>0.05).

DM, dry matter; CP, crude protein; NDF, neutral detergent fiber; ADF, acid detergent fiber; WSC, water-soluble carbohydrates.

**Table 6:** Chemical composition of peanut vine silage treated with different additive combinations after 60 d of ensiling

Items	Silage time (d)	Groups					p-value

		CK3	M-EP	M-L	EP-L	M-EP-L	
DM	3	27.4±0.3^b^	28.2±0.2^a^	28.2±0.4^a^	27.1±0.2^b^	28.3±0.7^a^	0.002
	7	26.9±0.3^b^	27.5±0.2^a^	27.6±0.2^a^	26.8±0.2^b^	27.5±0.2^a^	<0.001
	14	27.3±1.5^a^	27.5±0.3^a^	27.6±0.8^a^	26.8±0.4^a^	27.3±1.9^a^	0.91
	30	26.3±0.5^b^	27.3±0.2^a^	27.2±0.6^a^	26.1±0.4^b^	27.2±0.4^a^	0.003
	60	26.5±0.6^a^	27.4±0.2^a^	27.1±1.3^a^	26.5±0.5^a^	27.5±0.4^a^	0.13
CP	3	13.5±0.3^b^	14.0±0.2^a^	13.7±0.1^ab^	13.9±0.1^ab^	13.9±0.3^ab^	0.06
	7	13.3±0.1^b^	13.7±0.2^a^	13.3±0.2^b^	13.6±0.2^a^	13.6±0.1^a^	0.01
	14	13.7±0.2^a^	13.6±0.2^ab^	13.8±0.1^a^	13.9±0.3^a^	13.3±0.3^b^	0.02
	30	14.4±0.3^a^	13.9±0.6^b^	14.0±0.1^ab^	14.2±0.4^ab^	14.5±0.2^a^	0.06
	60	14.3±0.0^ab^	13.4±0.1^ab^	13.9±0.2^b^	14.8±0.3^a^	14.2±0.6^b^	<0.001
NDF	3	40.1±0.9^b^	40.8±0.4^ab^	42.1±0.5^a^	42.2±1.2^a^	41.2±1.0^ab^	0.008
	7	41.4±1.4^b^	41.1±0.3^b^	43.1±1.4^ab^	44.3±2.3^a^	42.9±1.4^ab^	0.06
	14	44.3±1.3^ab^	42.9±1.8^b^	45.0±0.7^a^	45.0±0.7^a^	40.2±1.0^c^	<0.001
	30	43.8±1.0^a^	44.7±2.4^a^	43.6±1.7^a^	44.1±1.2^a^	43.4±0.8^a^	0.79
	60	43.9±0.6^a^	42.9±1.6^ab^	43.0±1.6^ab^	44.1±0.6^a^	41.3±1.0^b^	0.03
ADF	3	30.4±0.7^c^	31.0±0.4^bc^	33.3±0.8^a^	31.8±1.2^b^	31.0±0.9^bc^	0.002
	7	34.4±1.1^ab^	33.4±0.3^b^	34.4±2.0^ab^	36.3±1.7^a^	34.7±0.9^ab^	0.45
	14	36.3±1.3^ab^	34.6±1.7^bc^	36.9±0.8^a^	36.9±0.9^a^	33.3±0.6^c^	0.001
	30	35.0±1.2^a^	36.0±2.7^a^	35.2±1.2^a^	35.2±0.6^a^	34.2±0.8^a^	0.60
	60	34.6±0.8^a^	34.0±1.5^ab^	33.9±1.3^ab^	35.6±0.9^a^	32.2±1.1^b^	0.01
WSC	3	1.45±0.13^b^	1.64±0.07^a^	1.54±0.19^ab^	1.11±0.07^c^	1.73±0.09^a^	<0.001
	7	1.14±0.15^ab^	1.11±0.16^ab^	1.21±0.17^a^	0.87±0.07^b^	1.26±0.29^a^	0.07
	14	0.91±0.11^bc^	0.90±0.14^bc^	0.96±0.15^b^	0.75±0.12^c^	1.25±0.11^a^	<0.001
	30	0.76±0.06^bc^	0.72±0.14^bc^	0.88±0.10^ab^	0.63±0.11^c^	1.01±0.12^a^	0.002
	60	0.75±0.10^b^	0.84±0.10^b^	0.82±0.05^b^	0.53±0.08^c^	1.05±0.10^a^	<0.001

CK = control group without additives; M-EP = molasses+enzyme preparation; M-L = molasses+lactic acid bacteria; EP-L = enzyme preparation+lactic acid bacteria; M-EP-L = molasses+enzyme preparation+lactic acid bacteria.

Measured items are expressed on a DM basis.

a–c Within the same row, values with different lowercase letters indicate significant differences (p<0.05), while those with the same letter are not significantly different (p>0.05).

DM, dry matter; CP, crude protein; NDF, neutral detergent fiber; ADF, acid detergent fiber; WSC, water-soluble carbohydrates.

**Table 7:** Effects of feeding wrapped peanut vine silage on the growth performance of *Hu* sheep

Items	Test period (d)	Control group	Test group	SEM	p-value
Initial weight (kg)	0	31.5	31.7	0.6	0.73
Final weight (kg)	56	43.9	43.3	1.0	0.60
ADG (g/d)	1–28	233.7	212.5	11.4	0.12
	29–56	212.1	215.4	15.7	0.79
	1–56	221.6	214.2	11.7	0.55
ADMI (kg/d)	1–28	1.6	1.5	0.0	0.16
	29–56	1.7	1.6	0.4	0.39
	1–56	1.6	1.6	0.0	0.13
F/G	1–28	6.74	7.20	0.22	0.08
	29–56	7.96	7.66	0.64	0.67
	1–56	7.36	7.39	0.42	0.92

Control group = basal diet containing peanut vine hay; Test group = basal diet in which peanut vine silage.

Statistical significance was declared at p<0.05.

ADG, average daily gain; ADMI, average dry matter intake; F/G, feed-to-gain ratio.

**Table 8:** Effects of feeding wrapped peanut vine silage on blood biochemical parameters of *Hu* sheep after 56 d feeding trial

Items	Test periods (d)	Control group	Test group	SEM	p-value
TP (g/L)	0	70.18	71.31	3.13	0.72
	28	71.43	73.06	2.28	0.48
	56	69.63	70.10	2.15	0.54
ALB (g/L)	0	32.53	33.15	2.08	0.77
	28	36.68	37.31	1.85	0.74
	56	38.53	38.00	1.12	0.62
GLB (g/L)	0	37.65	38.16	4.39	0.91
	28	34.75	35.75	3.48	0.78
	56	31.10	33.03	2.88	0.52
ALB/GLB	0	0.91	0.92	0.13	0.95
	28	1.10	1.09	0.13	0.93
	56	1.26	1.20	0.12	0.60
BUN (mmol/L)	0	8.21	7.98	0.67	0.74
	28	8.67	7.85	0.59	0.17
	56	8.87^a^	7.73^b^	0.47	0.04
GLU (mmol/L)	0	4.40	4.72	0.43	0.46
	28	5.71	4.96	0.45	0.12
	56	4.94	5.13	0.26	0.49
CHO (mmol/L)	0	1.43	1.51	0.09	0.26
	28	1.61	1.67	0.16	0.70
	56	1.72	1.69	0.13	0.81
TG (mmol/L)	0	0.28	0.33	0.04	0.28
	28	0.29^a^	0.22^b^	0.03	0.02
	56	0.32^a^	0.24^b^	0.02	0.01
HDL-C (mmol/L)	0	0.91	1.01	0.06	0.13
	28	1.06	1.10	0.10	0.62
	56	1.09	1.11	0.08	0.76
LDL- C (mmol/L)	0	0.48	0.51	0.05	0.56
	28	0.57	0.59	0.07	0.73
	56	0.63	0.60	0.06	0.54

Control group = basal diet containing peanut vine hay; Test group = basal diet in which peanut vine silage.

Within the same row, values with different lowercase letters differ significantly (p<0.05), whereas values with the same letter do not differ significantly.

TP, total protein; ALB, albumin; GLB, globulin; ALB/GLB, albumin-to-globulin ratio; BUN, blood urea nitrogen; GLU, glucose; CHO, cholesterol; TG, triglycerides; HDL-C, high-density lipoprotein cholesterol; LDL-C, low-density lipoprotein cholesterol.

## Data Availability

Upon reasonable request, the datasets of this study can be available from the corresponding author.
